# Congenital Absence of the Lateral Incisors

**Published:** 1852-04

**Authors:** Sam. D. Muse


For the Dental Register of the West.

### CONGENITAL ABSENCE OF THE LATERAL INCISORS.

On examination of the teeth of Mrs. McJ. I was astonished to find that she was minus the lateral incisors. Upon inquiry I ascertained that the deficiency was common in her family. Mrs. McJ. informs me that the first case that she knew of in the family was in that of her grandfather, himself wanting these teeth; also seven out of twelve of his children, ten daughters and two sons, the sons minus; and five daughters. Mrs. McJ.'s father's family, were twelve daughters, four had this remarkable deficiency. Of the offsprings of her sisters the largest number are daughters, of whom at least, she thinks, one half present this remarkable characteristic. Of Mrs. McJ.'s family of five children two cases of the same character appear. You may perhaps suppose that the absence of these teeth constitutes a deformity. This, however, is not the case; nature has compensated to a very remarkable degree for this absence by furnishing the central incisors rather remarkably broad and well developed, and in most of the cases the centrals occupy the precise locality of the laterals, hence the space is greatest between the centrals. I communicate this as a matter of curiosity, not having seen any account of such an hereditary absence of these important organs. If you find this communication of sufficient interest to deserve a place in the Dental Register, you are at liberty to tranfer it to its columns.

Respectfully yours,

SAM. D. MUSE.

